# Beyond Infection and Malignancy: Diagnostic Pitfalls in Pulmonary Granulomatosis with Polyangiitis

**DOI:** 10.7759/cureus.101192

**Published:** 2026-01-09

**Authors:** Alan Wang, Humberto E Trejo Bittar, John Greene

**Affiliations:** 1 Osteopathic Medicine, Nova Southeastern University Dr. Kiran C. Patel College of Osteopathic Medicine, Clearwater, USA; 2 Thoracic Pathology, Moffitt Cancer Center, Tampa, USA; 3 Infectious Diseases, Moffitt Cancer Center, Tampa, USA

**Keywords:** antineutrophil cytoplasmic antibody (anca), cancer, diagnostic challenge, granulomatosis with polyangiitis (gpa), small-vessel vasculitis, wegener’s

## Abstract

Granulomatosis with polyangiitis (GPA) is a rare but potentially life-threatening antineutrophil cytoplasmic antibody (ANCA)-associated small-vessel vasculitis. It frequently presents with pulmonary manifestations that mimic infection or malignancy, leading to significant diagnostic delays. We describe three cases that illustrate the broad clinical, radiologic, and pathologic spectrum of GPA and highlight common diagnostic pitfalls. The first case involves a hypermetabolic cavitary lung mass initially suspected to represent primary lung cancer; the second highlights the development of lymphoproliferative malignancy in a patient with longstanding GPA receiving chronic immunosuppressive therapy; and the third demonstrates how partial responses to corticosteroids and empiric antibiotics can mask ongoing vasculitic activity, resulting in delayed diagnosis despite progressive cavitary lung disease. Across cases, nonspecific imaging findings, inconclusive or misleading biopsies, and delayed or incomplete serologic evaluation further complicated early recognition. This case series underscores that maintaining a high index of suspicion for GPA, together with a multidisciplinary approach to interpreting often ambiguous clinical, serologic, radiologic, and pathologic data, is critical to avoiding diagnostic delay and improving patient outcomes.

## Introduction

Granulomatosis with polyangiitis (GPA) is an uncommon but potentially life-threatening antineutrophil cytoplasmic antibody (ANCA)-associated small-vessel vasculitis characterized by necrotizing granulomatous inflammation of the respiratory tract and variable renal involvement. Circulating proteinase 3 (PR3)-ANCA is present in approximately 60-70% of cases, myeloperoxidase (MPO)-ANCA in approximately 20%, and 10-20% of patients remain ANCA-negative [1]. Pathophysiologically, PR3-ANCA and MPO-ANCA activate primed neutrophils, driving small-vessel inflammation and endothelial injury that can damage capillary beds in the lungs and kidneys, resulting in manifestations such as pulmonary nodules, alveolar hemorrhage, and pauci-immune glomerulonephritis. GPA can occur at any age but most commonly affects older adults, with peak incidence between 65 and 74 years [2].

Clinical presentation is often nonspecific, contributing to diagnostic difficulty. Patients frequently report constitutional symptoms such as fever, fatigue, and weight loss, along with organ-specific manifestations including chronic sinusitis, otitis media, cough, hemoptysis, wheezing, or stridor [3]. Renal involvement, most commonly pauci-immune necrotizing crescentic glomerulonephritis, is present in only 10-20% of patients at initial presentation but develops in approximately 70-85% over the course of the disease [2]. Despite advances in immunosuppressive therapy, GPA remains a chronic relapsing condition, with recurrence reported in over 50% of patients and cumulative relapse rates approaching 40-60% within three to five years [4].

Pulmonary involvement is particularly common, affecting up to 80-90% of patients over the course of the disease and often presenting as bilateral pulmonary nodules or masses, frequently cavitary, as well as alveolar hemorrhage or other radiographic abnormalities that closely resemble infectious or malignant processes [2,5]. Because these thoracic findings overlap substantially with lung cancer, metastases, and granulomatous infections, GPA is frequently misdiagnosed. Indeed, up to 73% of patients with systemic vasculitis initially receive an incorrect diagnosis, most commonly infection, malignancy, or another autoimmune disorder, and diagnostic delays exceeding six months occur in approximately one-third of cases, particularly among older adults in whom classic otolaryngologic features may be subtle or absent [2,6].

Although tissue biopsy demonstrating necrotizing granulomatous inflammation and vasculitis remains the diagnostic gold standard [6], early samples may be inconclusive, and ANCA serologies may be negative or delayed [2]. Thoracic computed tomography (CT) can provide important diagnostic clues, including multiple nodules or masses, cavitary lesions, and patchy consolidation. Still, no single clinical, serologic, radiologic, or histopathologic feature is definitive in isolation [2,6,7]. As a result, accurate diagnosis often requires careful longitudinal integration of data and expert pathologic interpretation.

Standard induction therapy for GPA consists of high-dose corticosteroids combined with either cyclophosphamide or rituximab, both of which have significantly improved survival [5,8]. Randomized trials have demonstrated that rituximab is non-inferior to cyclophosphamide for remission induction, with particular benefit in PR3-ANCA-positive disease and a more favorable long-term toxicity profile [8]. Following remission induction, maintenance therapy with azathioprine, rituximab, or methotrexate is typically continued for 12-36 months to reduce relapse risk [8]. More recently, the C5a receptor inhibitor avacopan has emerged as an effective steroid-sparing option, reducing glucocorticoid exposure and associated toxicity while maintaining disease control [9]. Nevertheless, prolonged immunosuppression remains associated with serious complications, including infections and malignancies, reflecting both treatment-related carcinogenicity and disease-associated immune dysregulation [10,11].

This case series illustrates how these diagnostic and therapeutic challenges manifest in clinical practice. We present three cases that highlight key pitfalls in pulmonary GPA: (1) misdiagnosis as primary lung malignancy due to a hypermetabolic cavitary mass; (2) development of high-grade B-cell lymphoma in the setting of longstanding immunosuppression; and (3) prolonged diagnostic delay driven by partial, transient responses to corticosteroids and empiric therapy. Together, these cases emphasize the need for heightened clinical suspicion and multidisciplinary evaluation when GPA presents with atypical or malignancy-mimicking pulmonary features.

## Case presentation

Case 1

Clinical Timeline

In October 2021, there was an onset of cough and hemoptysis, and the patient was nonresponsive to antibiotics. In 2022, a hypermetabolic cavitary lung mass was biopsied, and GPA was diagnosed and treated.

Presentation

A 75-year-old Caucasian man with multiple comorbidities, including inflammatory polyarthritis on chronic low-dose prednisone, presented in 2022 for evaluation of granulomatous nodular pneumonia identified on prior imaging. Beginning in October 2021, he developed a persistent cough with intermittent hemoptysis refractory to azithromycin.

Investigations

**Figure 1:**
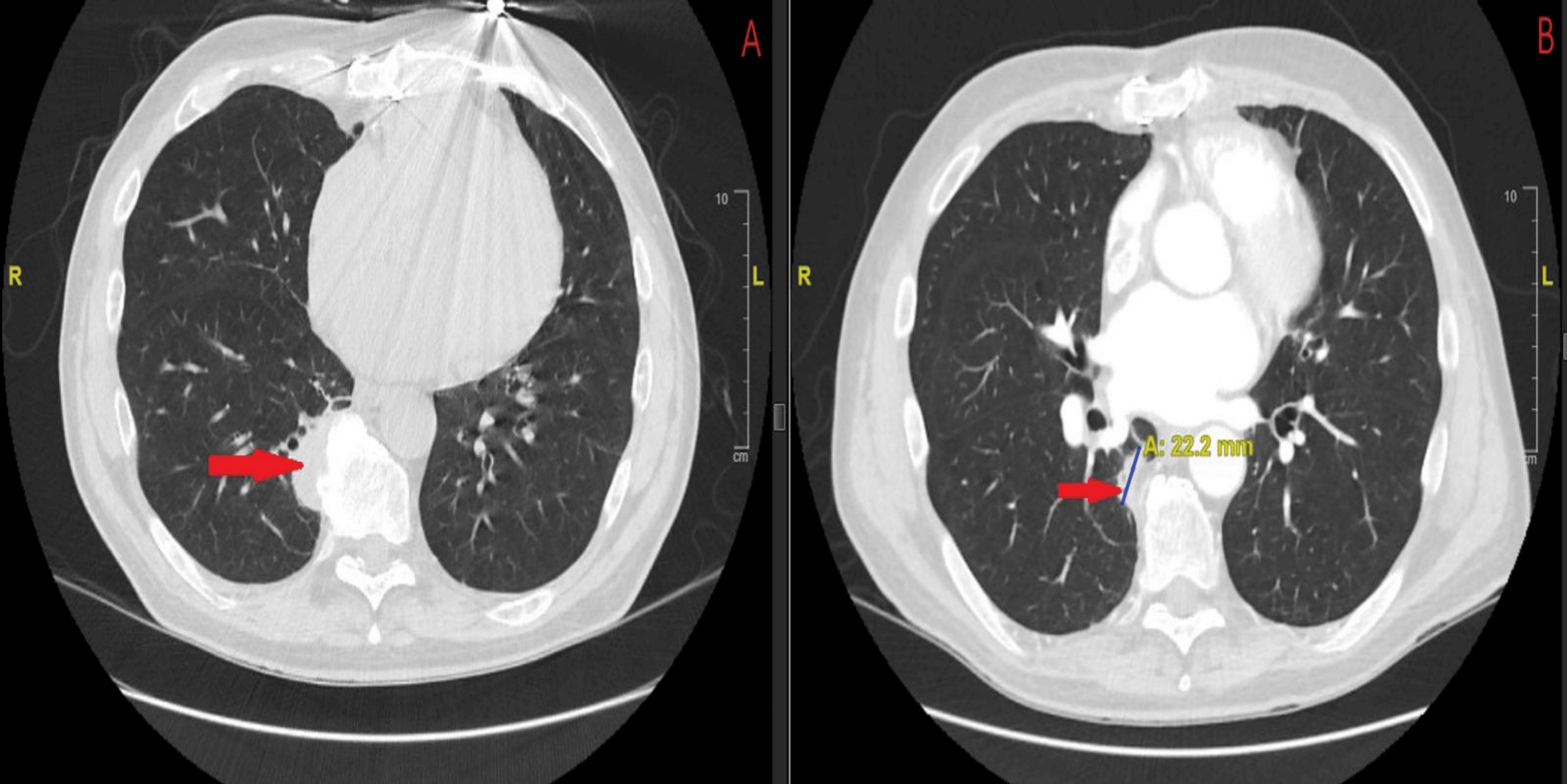
Axial contrast-enhanced CT of the chest demonstrating a pleural-based right lower lobe mass with irregular margins and partial cavitation (arrows). The lesion abuts the posterior pleura and spinous process and corresponded to a hypermetabolic focus on PET imaging. CT: computed tomography; PET: positron emission tomography

Chest CT demonstrated a progressively enlarging, pleural-based right lower lobe mass abutting the posterior pleura and spinous process, with irregular borders and partial cavitation (Figure 1). PET imaging revealed intense hypermetabolic activity, raising concern for malignancy.

**Figure 2:**
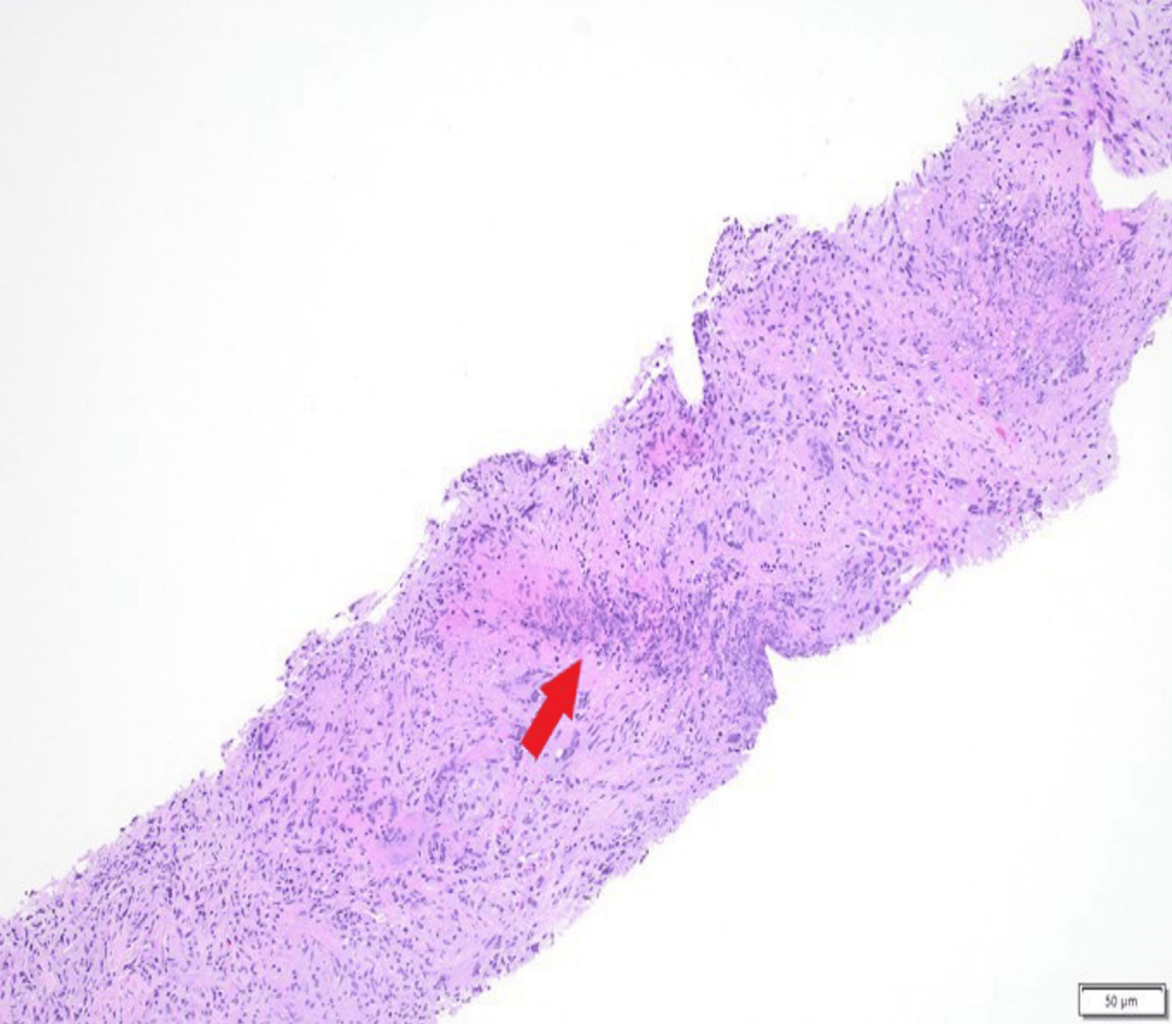
Histopathology showing necrotizing granulomatous inflammation with mixed inflammatory infiltrates and basophilic geographic necrosis (arrow) (hematoxylin and eosin stain). Scale bar: 50 µm

**Figure 3:**
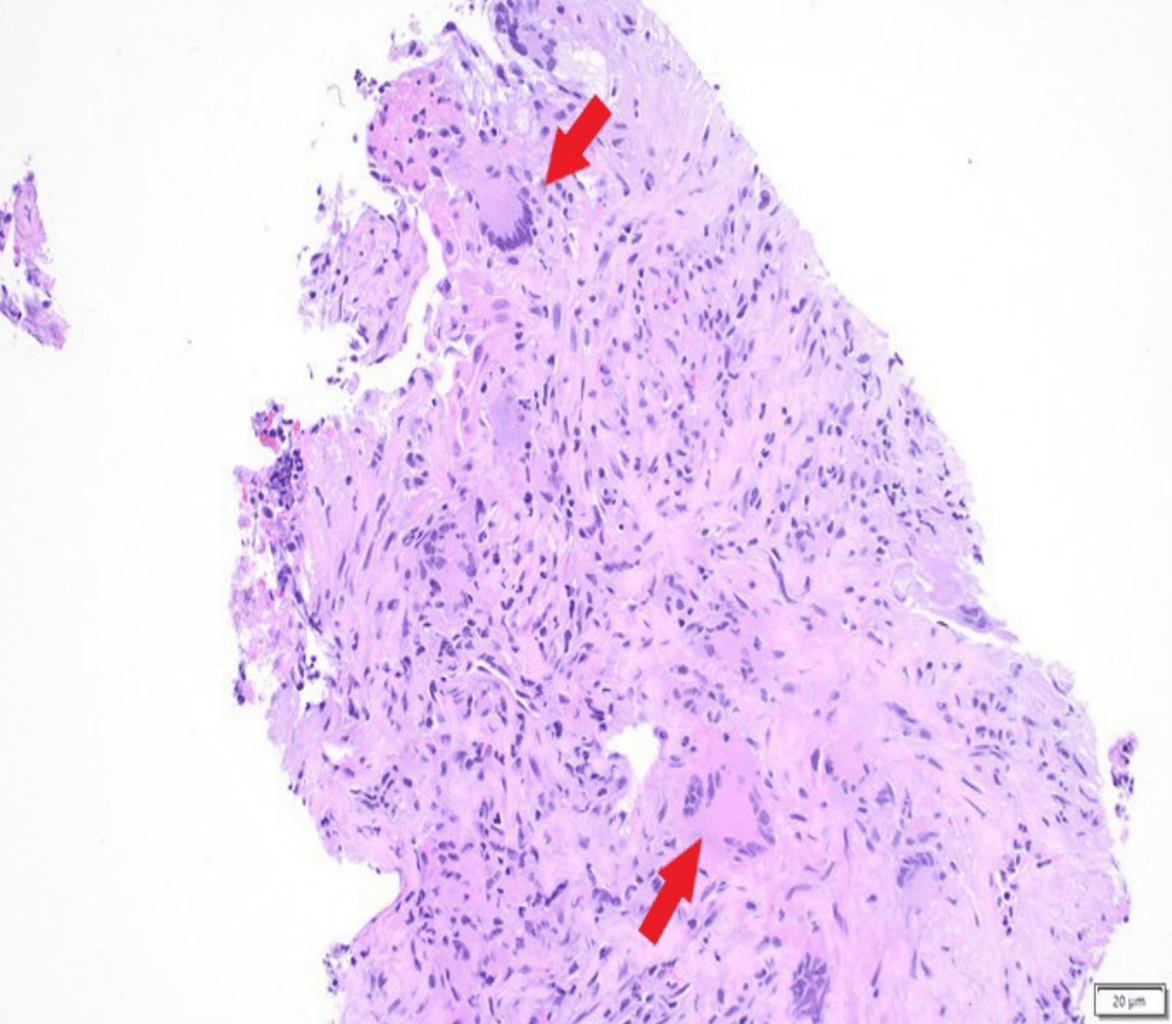
Granulomatous inflammation with prominent multinucleated giant cells (arrows) (hematoxylin and eosin stain). Scale bar: 20 µm

**Figure 4:**
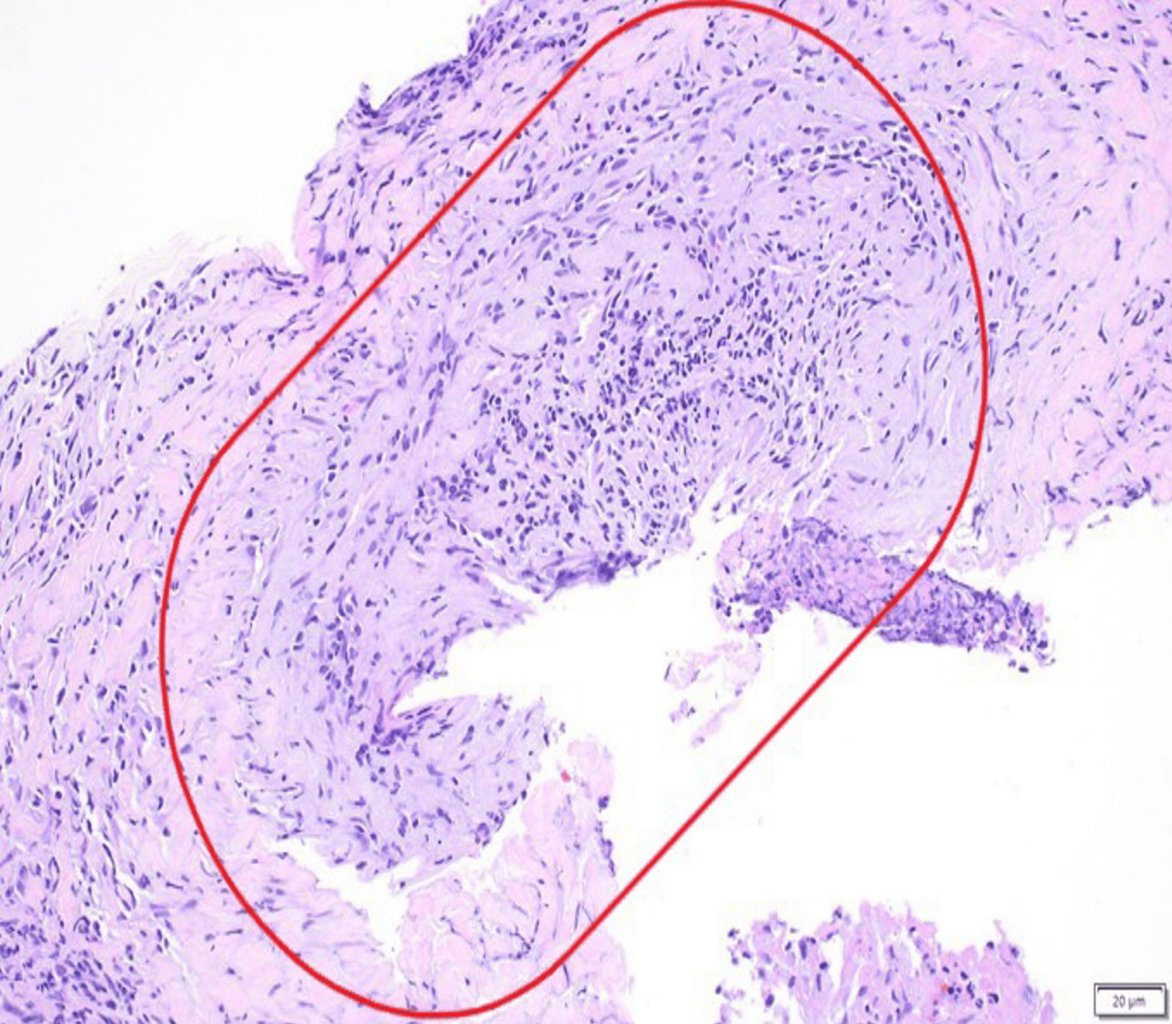
Focal small-vessel vasculitis identified within the biopsy specimen (circle), a finding that may be subtle in limited tissue samples (hematoxylin and eosin stain). Scale bar: 20 µm

CT-guided core needle biopsy demonstrated necrotizing granulomatous inflammation. Histopathologic examination revealed mixed inflammatory infiltrates with multinucleated giant cells, geographic necrosis, and focal small-vessel vasculitis (Figures 2-4). Special stains and cultures were negative for infectious organisms, and immunohistochemistry was negative for carcinoma.

Laboratory testing showed elevated PR3-ANCA, elevated inflammatory markers, and negative MPO-ANCA (Table 1).

**Table 1:** Laboratory results for Case 1 with corresponding reference ranges CRP: C-reactive protein; ESR: Erythrocyte sedimentation rate

Laboratory test	Patient value	Reference range
c-ANCA (PR3-ANCA)	4.6 U/mL	<3.5 U/mL
p-ANCA (MPO-ANCA)	Negative	Negative
CRP	12.5 mg/L	<5 mg/L
ESR	22 mm/hr	0–20 mm/hr (men)

Diagnosis

The diagnosis was pulmonary GPA.

Treatment

The patient was initiated on high-dose corticosteroids followed by rituximab induction therapy.

Outcome

Following induction therapy, cough and hemoptysis gradually resolved over several weeks. Inflammatory markers normalized, and interval chest imaging at three months demonstrated a substantial qualitative reduction in lesion size with near-complete resolution of surrounding inflammatory changes. Formal volumetric or percentage-based measurements were unavailable; however, the radiographic response was considered marked by both radiology and treating clinicians. He was transitioned to a tapering corticosteroid regimen and maintenance rituximab therapy, administered at regular intervals and guided by clinical response and B-cell repopulation. He remained clinically stable, with no recurrence of respiratory symptoms or new radiographic abnormalities.

Case 2

Clinical Timeline

In 2015-2021, GPA was stable on azathioprine. In 2021, new systemic symptoms were observed; a peritoneal mass was diagnosed as high-grade B-cell lymphoma.

Presentation

A 76-year-old woman with a history of GPA diagnosed in 2015, hypertension, coronary artery disease, chronic kidney disease, and interstitial lung disease presented in 2021 for evaluation of a newly diagnosed high-grade B-cell non-Hodgkin lymphoma. She had been maintained on azathioprine with stable pulmonary disease and good functional status. Several weeks prior to presentation, she developed progressive fatigue, early satiety, right-sided abdominal discomfort, unintentional weight loss of approximately 10 pounds, and worsening dyspnea beyond baseline.

Investigations

Laboratory evaluation revealed mild normocytic anemia (hemoglobin 11.9 g/dL; reference range 12-16 g/dL) and elevated lactate dehydrogenase (400 U/L; reference range 140-280 U/L). CT imaging of the abdomen and pelvis demonstrated a peritoneal mass. Core needle biopsy revealed sheets of medium- to large-sized atypical lymphoid cells positive for CD20 and CD10 and negative for BCL2. Proliferative indices were markedly elevated, with Ki-67 labeling in >95% of tumor cells and diffuse c-MYC protein expression in >95%, indicating a highly aggressive growth fraction. No fluorescence in situ hybridization (FISH) studies were available to assess MYC gene rearrangements.

**Figure 5:**
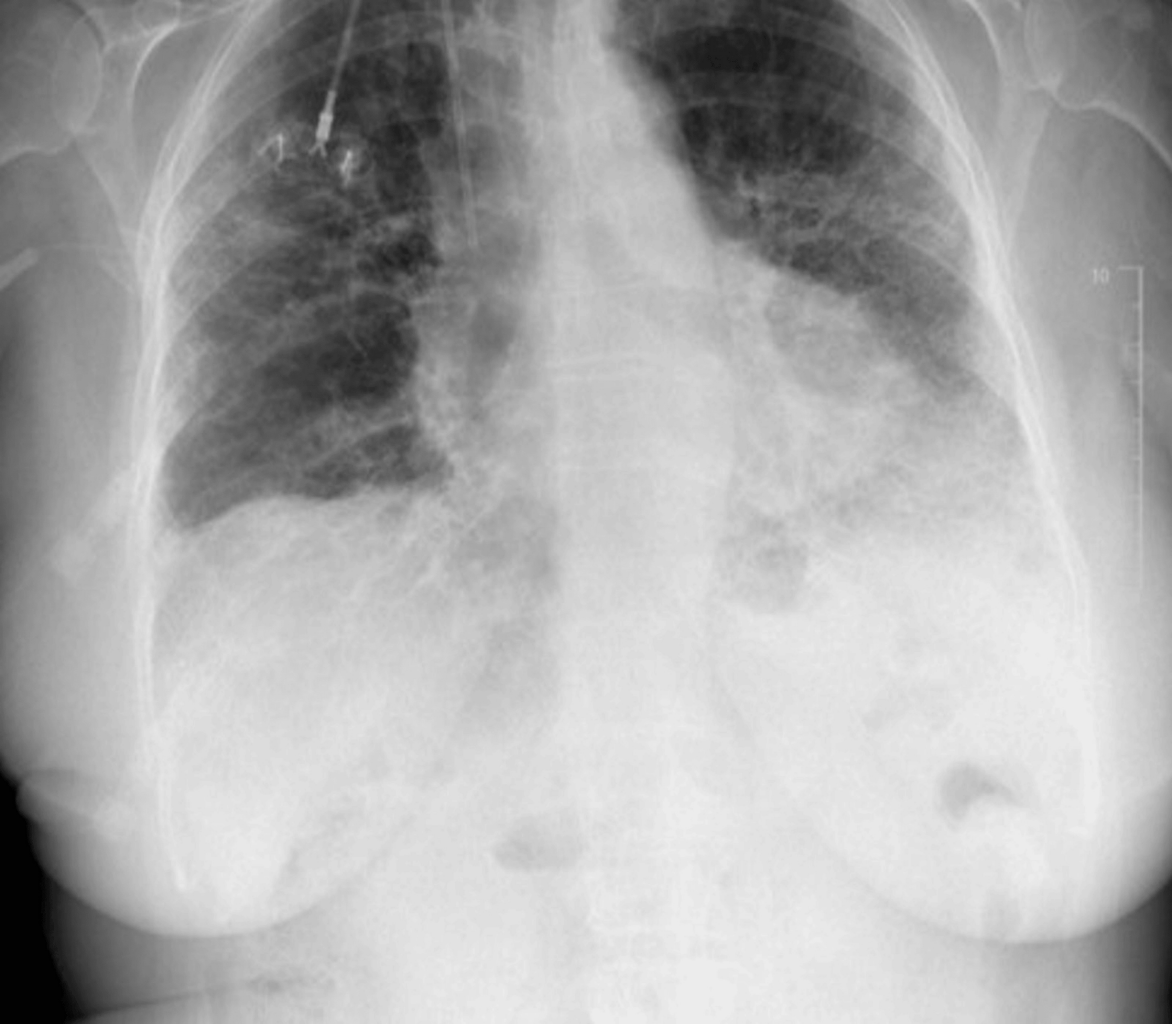
Chest radiograph demonstrating bilateral reticulonodular opacities and patchy consolidation consistent with chronic interstitial lung disease related to GPA. GPA: granulomatosis with polyangiitis

**Figure 6:**
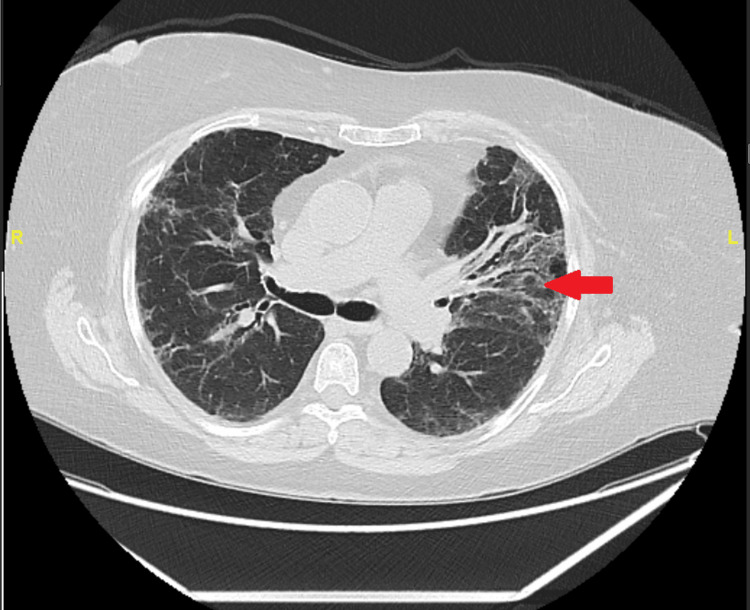
Contrast-enhanced CT of the chest showing fibrotic changes with architectural distortion and traction bronchiectasis, without new cavitary lesions or evidence of active pulmonary vasculitis. CT: computed tomography

Chest radiography and CT showed chronic interstitial lung disease without evidence of active pulmonary vasculitis (Figures 5, 6).

Diagnosis

The diagnosis was a high-grade B-cell non-Hodgkin lymphoma arising in a patient with longstanding GPA.

Treatment

Azathioprine was discontinued. The patient underwent staging evaluation, including PET-CT and bone marrow biopsy, and chemotherapy planning was initiated, with regimen selection dependent on final histologic classification.

Outcome

Pulmonary GPA remained clinically and radiographically stable at the time of lymphoma diagnosis.

Case 3

Clinical Timeline

In August 2007, the initial presentation was chronic cough and dyspnea, treated as cryptogenic organizing pneumonia. In November 2007, partial symptomatic improvement was observed after corticosteroids and azithromycin. In February 2008, there was a recurrence of cough and hemoptysis; imaging showed progression of cavitary nodules. In March 2008, PR3-ANCA positivity was found, and a Mayo Clinic pathology review confirmed GPA. In August 2008, marked radiographic and functional improvement was observed after immunosuppressive therapy.

Presentation

A 57-year-old woman with a remote history of pneumonia approximately 30 years earlier was referred in August 2007 for evaluation of presumed pneumonia. She had been in good health until six months prior, when she developed a persistent cough and progressively worsening dyspnea. Initial symptomatic improvement was achieved with an inhaler and benzonatate, but symptoms subsequently worsened.

Investigations

Chest imaging revealed multiple large bilateral pulmonary nodules, several with cavitation and associated consolidation. PET-CT demonstrated increased metabolic activity without mediastinal or hilar lymphadenopathy. Bronchoscopy with bronchoalveolar lavage and cultures for bacterial, fungal, and mycobacterial organisms were negative. Wedge resection of lung tissue demonstrated organizing pneumonia with microabscess formation; special stains for acid-fast bacilli and fungi were negative. Interval CT imaging showed progression of nodular infiltrates with central necrosis.

The patient recalled prior respiratory cultures yielding *Pseudomonas aeruginosa*, *Candida glabrata*, and coagulase-negative *Staphylococcus*, for which she was treated with intravenous ceftriaxone and levofloxacin.

Diagnosis

The diagnosis was initially presumed cryptogenic organizing pneumonia, later confirmed as pulmonary GPA.

Treatment

Following her initial presentation, she was treated with azithromycin 250 mg daily for three months and a short prednisone taper. Despite transient improvement, symptoms persisted, and imaging demonstrated progression of cavitary nodules. Empiric therapy with higher-dose corticosteroids and levofloxacin was initiated.

At a March 2008 follow-up, serologic testing revealed elevated PR3 antibodies with positive c-ANCA. The original biopsy specimens were reviewed at Mayo Clinic and demonstrated necrotizing granulomatous inflammation with vasculitis. She was subsequently initiated on induction therapy with corticosteroids and cyclophosphamide.

Outcome

**Figure 7:**
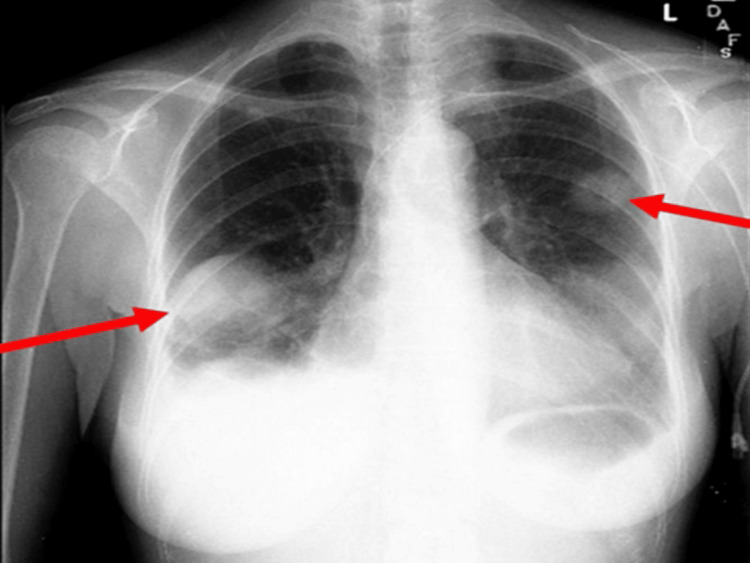
Chest radiograph demonstrating multiple bilateral cavitary pulmonary nodules with surrounding consolidation (arrows).

**Figure 8:**
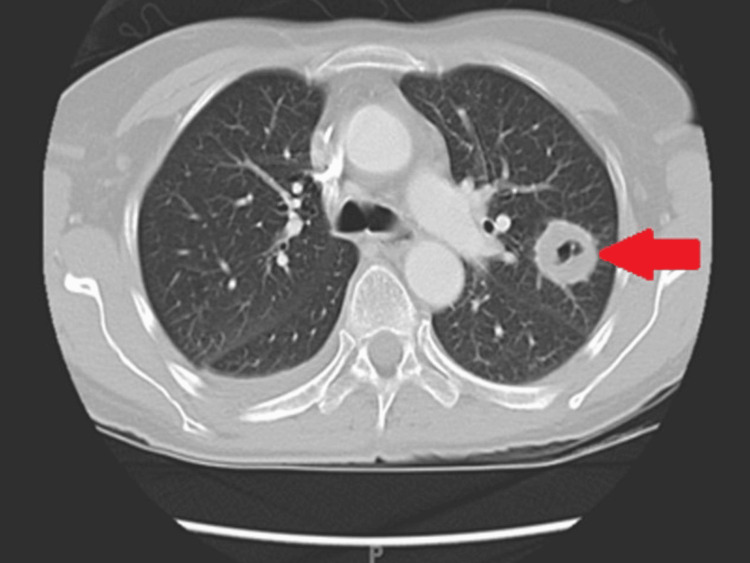
Contrast-enhanced CT of the chest showing a cavitary nodule with irregular margins, corresponding to necrotizing granulomatous inflammation confirmed on histopathology. CT: computed tomography

At follow-up in August 2008, chest CT showed a marked reduction in the size and number of pulmonary nodules (Figures 7, 8). Pulmonary function testing demonstrated significant improvement, with total lung capacity increasing from 69% to 91% predicted. A definitive diagnosis of GPA was established on the basis of clinical, serologic, radiologic, and histopathologic findings.

Summary of Cases

To facilitate comparison and improve clarity, the key clinical, radiologic, pathologic, and therapeutic features of all three cases are summarized in Table 2. This table highlights patient demographics, presenting symptoms, salient imaging findings, biopsy results, ANCA status, initial working diagnoses, final diagnoses, and clinical outcomes. Together, these cases illustrate the broad spectrum of pulmonary involvement in GPA, its ability to mimic infection and malignancy, and the diagnostic and therapeutic challenges encountered across clinical contexts.

**Table 2:** Summary of clinical features across three cases of GPA GPA: granulomatosis with polyangiitis; RLL: right lower lobe; CT: computed tomography; PET: positron emission tomography; PR3-ANCA: proteinase 3-anti-neutrophil cytoplasmic antibody

Patient demographics	Key presenting symptoms / signs	Salient imaging findings	Biopsy results	ANCA status	Initial misdiagnosis	Final diagnosis	Treatment / outcome
Case 1: 75-year-old male	Chronic cough, intermittent hemoptysis	Hypermetabolic pleural-based cavitary RLL mass on CT/PET	Necrotizing granulomatous inflammation with multinucleated giant cells and focal small-vessel vasculitis	PR3-ANCA positive	Primary lung malignancy	Pulmonary GPA	High-dose corticosteroids + rituximab induction → maintenance rituximab; clinical and radiographic remission
Case 2: 76-year-old female	Fatigue, weight loss, abdominal discomfort	Stable fibrotic lung disease; peritoneal mass on CT	High-grade B-cell lymphoma (CD20+, CD10+, Ki-67 >95%)	Not available (prior GPA diagnosis)	Not applicable (known GPA; new malignancy workup)	Secondary lymphoma in GPA	Discontinuation of azathioprine; oncologic staging and chemotherapy planning
Case 3: 57-year-old female	Chronic cough, dyspnea, recurrent hemoptysis	Multiple enlarging cavitary pulmonary nodules with PET avidity	Necrotizing granulomatous inflammation with vasculitis (Mayo Clinic review)	PR3-ANCA positive	Cryptogenic organizing pneumonia	Pulmonary GPA	Corticosteroids + cyclophosphamide induction → marked clinical, radiographic, and functional improvement

## Discussion

The clinical and radiologic diversity of GPA makes it one of the most challenging systemic vasculitis to diagnose, a complexity clearly illustrated by all three cases in this series. The first-line diagnostic evaluation typically begins with ANCA testing, and recent consensus guidelines recommend enzyme-linked immunosorbent assay (ELISA) as the preferred initial modality [12]. Although ANCA negativity is a well-recognized diagnostic pitfall in GPA, our cases highlight a related but distinct challenge: diagnostic delay despite eventual ANCA positivity or established disease history. This was most evident in Case 3, in which initial serologic evaluation was unrevealing or not pursued early, and a definitive diagnosis required repeated histopathologic review and delayed demonstration of PR3-ANCA positivity. In contrast, Case 1 demonstrated PR3-ANCA positivity at presentation; however, diagnosis was delayed due to strong radiographic concerns for malignancy. In Case 2, ANCA status was not central at presentation, given a pre-existing diagnosis of GPA. Taken together, Case 1 highlights malignancy mimicry and the limits of PET avidity; Case 2 underscores the risk of malignancy during long-term immunosuppression; and Case 3 illustrates how transient steroid responsiveness can delay definitive diagnosis.

GPA is also well known for mimicking lung cancer, particularly when pulmonary nodules cavitate, are peripherally located, or demonstrate intense 18F-fluorodeoxyglucose (FDG) uptake on PET, features prominently seen in Case 1, where the patient was explicitly referred for malignancy evaluation. Such presentations are well documented: up to 25% of GPA nodules larger than 2 cm cavitate; PET avidity is common; and radiographic and metabolic characteristics frequently overlap with those of malignant tumors [8]. Emerging data suggest that inflammatory cavitary lesions in GPA may demonstrate intense FDG uptake with ill-defined margins and central necrosis, limiting the specificity of PET imaging and reinforcing the need for tissue diagnosis in such cases. Even biopsy specimens may be misleading early in the disease course, as necrotizing granulomas can resemble carcinoma or infection when vasculitic features are subtle or absent. Recognizing these malignant mimics is especially important in older adults, such as in Case 1, where pulmonary-limited disease occurred without classic ear, nose, and throat (ENT) or renal involvement and therefore fell outside the traditional clinical profile [2].

In addition to radiologic features suggestive of malignancy, the pulmonary manifestations of GPA are highly nonspecific, further complicating diagnosis. Patients commonly present with cough, dyspnea, chest pain, or hemoptysis, while chest radiographs and CT scans may show unilateral or bilateral nodules with cavitation in up to half of cases [3,13], as demonstrated in Cases 1 and 3. Pulmonary infiltrates may occur, bronchoscopy may reveal airway narrowing due to granulomatous inflammation, and GPA may produce pseudotumoral lung masses that closely resemble neoplasms. Alveolar hemorrhage represents one of the most severe pulmonary manifestations and may progress to acute respiratory distress syndrome. When pulmonary involvement coincides with acute kidney injury, the classic pulmonary-renal syndrome emerges. Depending on the organs involved, ultrasonography, CT, or magnetic resonance imaging (MRI) may aid lesion characterization; however, imaging findings are often insufficient to differentiate vasculitis from infection or malignancy reliably [14]. FDG-PET, widely used in oncologic and inflammatory disease evaluation, has demonstrated superior diagnostic performance compared with gallium scanning, with reported sensitivity and specificity of approximately 80.2% and 89.8%, respectively, versus 60% and 63% for gallium imaging [15]. Nevertheless, FDG uptake patterns in GPA frequently mimic those of malignant lesions, as illustrated in Case 1, underscoring the limited specificity of PET imaging in isolation [16].

FDG-PET/CT has been shown to be highly sensitive for detecting active disease in GPA, particularly in pulmonary and sinonasal involvement, and may identify inflammatory lesions not yet apparent on conventional CT. Studies suggest that GPA-related cavitary lesions often demonstrate intense FDG uptake with ill-defined margins and central necrosis, features that substantially overlap with those of malignant and infectious processes [17]. Although FDG uptake intensity may correlate with disease activity and can assist in identifying sites for biopsy, it does not reliably distinguish vasculitic inflammation from malignancy. This limitation was clearly demonstrated in Case 1, where marked FDG avidity of a cavitary lung mass closely mimicked primary lung cancer and necessitated histopathologic confirmation. Despite these diagnostic challenges, outcomes improve substantially with appropriate therapy: untreated GPA carries a nearly 70% one-year mortality rate, whereas over 80% of patients achieve remission with treatment, and 10-year survival approaches 75% [13]. ENT involvement is generally associated with a better prognosis, although it is also linked to higher relapse rates.

Compounding diagnostic uncertainty is the increased risk of malignancy inherent to GPA and further amplified by its treatment. Cyclophosphamide, historically a cornerstone of induction therapy, is well established to increase the risk of secondary malignancies, including bladder cancer, leukemia, and lymphomas, particularly at cumulative doses exceeding 36 g [18]. Although generally considered safer, azathioprine has also been associated with lymphoproliferative disease in susceptible patients [19]. Beyond treatment-related carcinogenesis, GPA itself is characterized by chronic immune dysregulation and sustained inflammatory activity, which may contribute to oncogenesis through persistent endothelial injury, impaired immune surveillance, and a pro-tumorigenic inflammatory milieu driven by reactive oxygen species (ROS), cytokines, and aberrant neutrophil and lymphocyte activation [9,19]. These disease- and treatment-related factors likely act synergistically and are clinically illustrated by Case 2, in which a patient with longstanding GPA and prolonged immunosuppressive exposure developed high-grade B-cell lymphoma. Importantly, distinguishing malignancy from vasculitic relapse remains particularly challenging, as both processes can present with overlapping systemic symptoms, inflammatory markers, and imaging abnormalities, underscoring the need for careful staging and expert pathologic evaluation when new systemic or abdominal findings emerge in patients with GPA.

In recent years, treatment paradigms for GPA have evolved substantially in response to concerns regarding cyclophosphamide-related toxicity. Rituximab has largely replaced cyclophosphamide as the preferred first-line agent for remission induction, particularly in relapsing disease [13]. Randomized trials and systematic reviews have demonstrated that rituximab is non-inferior to cyclophosphamide for remission induction in ANCA-associated vasculitis, with comparable overall efficacy and particular benefit in PR3-ANCA-positive GPA. This evidence directly informed the management of Case 1, in which rituximab-based induction led to rapid clinical and radiographic improvement. Emerging data suggest rituximab may be superior in selected populations, with one weighted analysis reporting 6-month remission rates exceeding 70% compared with approximately 40% among cyclophosphamide-treated patients [13]. Reflecting this evidence, the 2021 American College of Rheumatology/Vasculitis Foundation guideline conditionally recommends rituximab over cyclophosphamide for induction therapy in active, severe GPA, largely due to its more favorable long-term safety profile, including a reduced risk of treatment-related malignancy [20].

The availability of newer targeted therapies further underscores the importance of individualized treatment selection in GPA, particularly for patients at heightened risk of treatment-related toxicity. In the context of this case series, steroid-sparing strategies are especially relevant to Cases 2 and 3, in which prolonged corticosteroid exposure contributed to cumulative immunosuppression and diagnostic masking, respectively. Agents such as avacopan may reduce cumulative glucocorticoid exposure, which may be particularly beneficial in patients with relapsing disease, prolonged treatment courses, or increased susceptibility to infectious and metabolic complications. Although avacopan was unavailable during the management of Cases 2 and 3, its use today might mitigate the risk of malignancy in patients like Case 2 and reduce steroid-driven diagnostic delay, as seen in Case 3. Maintenance regimens with azathioprine, rituximab, or methotrexate remain standard practice. Importantly, contemporary guidelines now conditionally recommend against the routine use of plasma exchange for remission induction in patients with GPA and active glomerulonephritis, reflecting evidence that it does not confer a clear survival or renal benefit in most cases [20]. Plasma exchange may still be considered selectively in patients with rapidly progressive renal failure on a case-by-case basis.

Diagnosis becomes even more challenging when patients exhibit partial, transient improvement with corticosteroids or immunomodulatory antibiotics. Corticosteroids rapidly suppress inflammation regardless of etiology, potentially masking ongoing vasculitic injury. More than one-third of patients with GPA experience diagnostic delays exceeding 1 year, often because empirical therapy temporarily slows disease progression without addressing the underlying process [6]. This phenomenon was clearly demonstrated in Case 3, where repeated courses of prednisone and azithromycin produced brief symptomatic improvement, initially suggesting organizing pneumonia or atypical infection. At the same time, cavitary lung lesions continued to progress. A definitive diagnosis was achieved only after expert histopathologic review confirmed necrotizing granulomatous vasculitis, underscoring the need to maintain a high index of suspicion despite apparent clinical improvement.

Finally, interpreting new pulmonary findings in patients with known GPA remains a significant clinical challenge. Chronic vasculitic damage, fibrosis, susceptibility to opportunistic infections, treatment-related toxicity, and the intrinsic malignancy risk associated with both the disease and its therapy contribute to a complex diagnostic landscape. As illustrated in Cases 2 and 3, radiographic patterns alone rarely distinguish among vasculitic relapse, infection, organizing pneumonia, and malignancy. Cultures may be negative, and biopsies may show nonspecific granulomatous inflammation without definitive vasculitis. Expert pathology review, serial imaging, and multidisciplinary collaboration are therefore essential. These cases reinforce the importance of considering GPA in the differential diagnosis of cavitary lung lesions, whether in newly referred patients or in those with established disease who develop new pulmonary abnormalities.

This study has several limitations inherent to its design. Because this is a small retrospective case series from a tertiary referral center, the findings are subject to selection bias and may overrepresent diagnostically complex or severe presentations of GPA. Quantitative imaging metrics, such as volumetric or percentage-based reduction in pulmonary nodules, were not uniformly available, limiting objective comparison of radiographic response across cases. In addition, the absence of standardized imaging protocols and longitudinal biomarker assessment restricts the ability to draw firm conclusions regarding treatment response. Despite these limitations, the cases were intentionally selected to highlight recurring diagnostic pitfalls and clinically relevant challenges that are frequently encountered in real-world practice but underrepresented in controlled trials.

## Conclusions

GPA remains a diagnostically challenging multisystem disease, particularly when pulmonary manifestations closely resemble infection or malignancy. As illustrated in Case 1, GPA may present as a hypermetabolic cavitary lung mass mimicking primary lung cancer; Case 2 demonstrates the increased risk of secondary malignancy associated with long-term immunosuppression in patients with established GPA; and Case 3 highlights how partial and transient steroid responsiveness can obscure the underlying vasculitic process and contribute to prolonged diagnostic delay. Together, these cases underscore the need for a high index of suspicion and careful integration of clinical, radiologic, serologic, and histopathologic data when evaluating atypical pulmonary findings.

Looking ahead, emerging diagnostic strategies, including more nuanced interpretation of PET-CT findings, expanded serologic profiling, and early expert pathologic review, may help reduce diagnostic delay in complex presentations such as those seen in Cases 1 and 3. In parallel, evolving therapeutic approaches, including B-cell-targeted therapies and steroid-sparing agents such as avacopan, may mitigate cumulative treatment-related toxicity and malignancy risk, particularly in patients like Case 2.

This case series is limited by its small sample size and retrospective design and may be subject to selection bias toward diagnostically complex presentations. Nonetheless, these cases highlight recurrent, clinically relevant pitfalls encountered in real-world practice and emphasize the importance of multidisciplinary collaboration to improve diagnostic accuracy and long-term outcomes in GPA.
